# Sero-prevalence of Hepatitis B and C at a Primary Health Care Facility in Ghana

**DOI:** 10.4314/ahs.v24i4.5

**Published:** 2024-12

**Authors:** Madison Adanusa, George Adjei, Sebastian Eliason, Samuel Amoah, Benson Cecil, Ignatius Sirikyi, Faustina Pappoe, Anasthasia Ofori, Frederick Adjei, Bernice Appau, Benjamin Nyane, Arthur Rudolf, Hagan Oheneba

**Affiliations:** 1 University of Cape Coast, Directorate of University Health Services; 2 University of Cape Coast School of Medical Sciences, Department of Community Medicine; 3 University of Cape Coast School of Medical Sciences, School of Medical Sciences, Department of Community Medicine; 4 University of Cape Coast School of Medical Sciences, Department of Immunology and Microbiology; 5 University of Cape Coast School of Nursing and Midwifery; 6 University of Cape Coast School of Medical Sciences, Department of Medical Biochemistry

**Keywords:** Hepatitis B and C, Primary Health Care Facility, Ghana

## Abstract

**Background:**

The advent of emerging global infections such as SARS-CoV-2 has brought forth the public health crises of neglected diseases in the LMICs. Viral hepatitis infections remain a public health problem especially in the advent of emerging. Viral hepatitis which preponderantly afflicts citizens of LMICs is one such group of diseases which exerts considerable burden in these countries, especially, hepatitis B and C. There is an effective vaccine against Hepatitis B and curative treatment to hepatitis C, however, access has been hampered resulting in deleterious sequalae. Identifying population afflicted by these infections could lead to prevention of the complications.

**Materials and Methods:**

Retrospective review of electronic data on individuals screened for hepatitis B and C at the University of Cape Coast Hospital were abstracted. Data abstracted included hepatitis B and C test status, age, sex, previous immunisation history and region of residence. Overall prevalence and prevalence in different categories were calculated.

**Results:**

Data for 6,006 were collected and used for the analyses. The overall prevalence for hepatitis B in the study group was 5.06%. The prevalence for hepatitis C was 0.93%.

**Conclusion:**

The burden of hepatitis B and C infection in Ghana is still high.

## Introduction

The emergence of SARS-CoV-2 infection in 2019 and the unprecedented focus generated towards the disease (COVID-19), has once again highlighted the general neglect of the global community towards diseases of public health importance that disproportionately afflict nations in the lower and the middle-income bracket of the global economic spectrum^1^,^2^. Of these “neglected” infectious diseases of public health importance, the annual mortality for malaria, tuberculosis and HIV have been decreasing over the years whereas mortality attributable to viral hepatitis infection has been increasing^3^. Thus, viral hepatitis caused be hepatitis A, B, C, D and E remain a global public health challenge. Of the 1.4 million deaths attributable to viral hepatitis reported annually worldwide, about 95% result from infection with HBV and HCV mostly from complications such as liver fibrosis, hepatocellular carcinoma and liver cirrhosis^4^,^5^. Currently, it is estimated that 296 million individuals have HBV chronic infection globally and worryingly, only about 10% of these individuals are aware of their status with only approximately 22% of these individuals receiving therapy^6^. HCV infections rate have been on the decline due to increased treatment with direct acting antiretrovirals, however, an estimated 58 million people worldwide have chronic infection resulting from HCV infection^6^. The availability of an efficacious vaccine and disease modifying treatment for HBV and curative treatment for HCV spurred the WHO in 2016 to formulate policies aimed at reducing new infections by 95% and 80% in HBV and HCV respectively in addition to reducing mortality by 65% in both HBV and HCV by 2030.

In line with the WHO policy guidelines, the Ghanaian government introduced Ghana Viral Hepatitis Guidelines in 2016. The guideline advocates for health facilities to actively test for viral hepatitis, especially, HBV and HCV, followed by the treatment of eligible individuals^7^. Currently, the prevalence of HBV and HCV in Ghana have been estimated at 12.3% and 3% respectively^8^,^9^. With a current population of 30.8 million^10^, chronic carriage of HBV and HCV could potentially afflict 3.8 million and 900 thousand of the population respectively. Majority of these individuals would not be aware of their infection status due to inaccessibility to testing regimes and other factors^11^. However, multiple barriers to diagnosis and treatment at the individual, community and the health-care facilities levels serve as impediments to the achievement of the elimination strategies^11^,^12^. The UCC Hospital regularly screens for HBV and HCV infection and this retrospective study was undertaken to determine the burden of disease within the screened population.

## Materials and methods

### Study design and site

The study was a retrospective review of patients' record at the University of Cape Coast Hospital. The hospital is a 400-bed primary health care facility which mainly caters to inhabitants of Cape Coast Municipality and adjoining districts^13^. The records included in the study were of patients who had been screened for HBV and HCV from July to December 2019. Ethical clearance for the study protocol was granted by the University of Cape Coast Institutional Review Board (UCCIRB/EXT/2020/2024) with a waiver of informed consent.

All male and female patients irrespective of their ages screened for HBV and HCV infection at the University of Cape Coast Hospital with complete required data in the hospital records at the time of the study were eligible to be included in the study.

### Data collection and analyses

Trained personnel extracted de-identified data from an electronic database of the hospital covering the study period. Data collected included patient age, sex, region of residence and birth, HBV and HCV serological result and HBV immunisation status (complete or incomplete). Data was extracted using the data collection tool, Epicollect 5 (Centre for Genomic Pathogen Surveillance, UK). Data in the comma-separated value (csv) format was downloaded from Epicollect 5 into Microsoft Excel worksheet (Microsoft Inc, USA). Data cleaning was performed to remove duplicate and incomplete data. Sero-prevalence was calculated as the percentage of participants with any type of infection for each sub-category. Pearson's chi-squared was utilised to determine the association between participant characteristics and infection status. Multivariable logistics modelling was used to determine the risk factors associated with infection status. The results were expressed as Odds ratios with 95% confidence interval. A p-value of less than 0.05 was considered significant for all the statistical analyses. All statistical analyses were undertaken using GraphPad Prism 9 (GraphPad Software Inc., USA) and the STATA 14 statistical analytical tool (Stata Corporation, USA). The prevalence map was rendered using the IBM Watson Studio Legacy Map tool (IBM, USA).

## Results

A total of 6,058 individual patient data were abstracted for the study out of which 6,006, that is approximately 99% were complete for sex, age, HBV vaccination status, region of residence, HBV and HCV serology results. The mean age of the 6,006 patients was 21.54 (± 5.27) ranging between 6 to 73 years. Of the 6006, 56.16% (3193) were males with a mean age of 22.33 years (± 5.68 years) ranging between 6 to 73 years, whereas the females made up 43.84% (2813) with a mean age of 20.64 (± 4.60) ranging between 10-45 years. The highest proportion of the participants resided in Central, Greater Accra and Ashanti Regions, that is, 29.92% (1797), 22.63% (1359) and 15.63% (939) respectively.

The proportion of patients who were born before the introduction of the Universal Childhood Vaccination (UCV) against HBV infection in 2002 was 4.38% (263), 68.06% (179) of whom reported no previous vaccination against HBV. Of the remaining 84 who had reported receiving vaccination previously, 35.71% (30) reported receiving 1 or 2 doses of the vaccine.

### Prevalence of HBV and HCV

The overall prevalence of HBV and HCV among the study participants was 5.06% and 0.93% respectively. The prevalence for HBV-HCV co-infection was estimated to be 83 per 100,000 participants. The regional distribution of the HBV and HCV prevalence according to participant region of residence in Ghana is shown in figure 1. Table 1 shows the prevalence of HBV and HCV in various categories.

**Figure:**
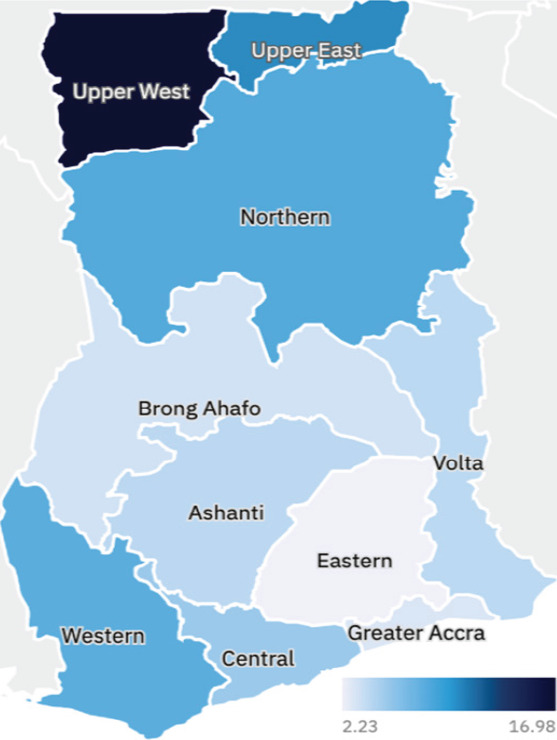


**Figure:**
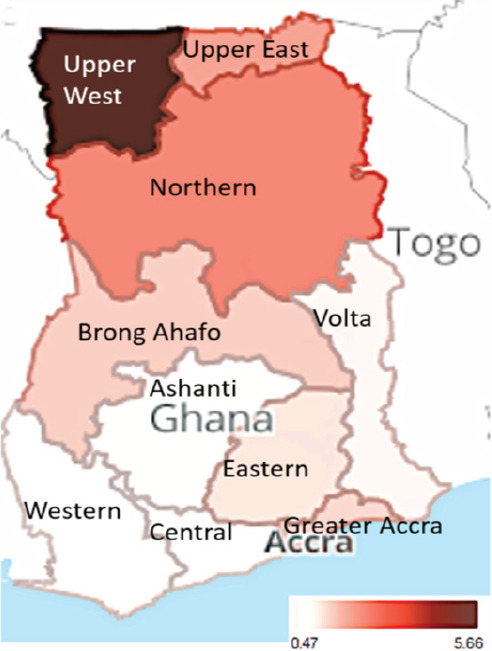


**Table:** 

		HBV				HCV			
Variable		COR (95% CI)	*p*-value	AOR (95% CI)	*p*-value	COR (95% CI)	*p*-value	AOR (95% CI)	*p*-value
Sex*	Female	1 (Ref)		1 (Ref)		1 (Ref)		1 (Ref)	
	Male	3.55 (2.38-5.29)	< 0.001	2.77 (1.84-4.15)	< 0.001	1.37 (0.80, 2.34)	0.257	1.18 (0.67, 2.08)	0.560
Age *	≤ 20	1 (Ref)		1 (Ref)		1 (Ref)		1 (Ref)	
	21-30	0.38 (0.30, 0.49)	0.000	0.48 (0.37, 0.62)	0.000	0.69 (0.40, 1.20)	0.187	0.89 (0.50 1.59)	0.691
	31-40	0.30 (0.19 0.47)	0.000	0.45 (0.28, 0.73)	0.001	0.64 (0.19, 2.13)	0.469	0.93 (0.27, 3.22)	0.913
	> 40	0.48 (0.22, 1.00)	0.050	0.68 (0.32, 1.45)	0.319	1.00 (0.13, 7.37)	0.997	1.36 (0.18, 10.35)	0.765
Region^*¿^	Central	1 (Ref)		1 (Ref)		1 (Ref)		1 (Ref)	
	Ashanti	1.31 (0.91, 1.89)	0.147	1.36 (0.94, 1.98)	0.104	1.70 (0.55, 5.24)	0.353	1.77 (0.57, 5.46)	0.322
	Brong Ahafo	1.49 (0.79, 2.81)	0.217	1.69 (0.89, 3.21)	0.106	0.28 (0.11, 0.71)	0.008	0.30 (0.12 0.75)	0.011
	Eastern	1.63 (0.94, 2.84)	0.081	1.74 (1.00, 3.04)	0.050	0.75 (0.24, 2.30)	0.611	0.77 (0.25, 2.38)	0.652
	Greater Accra	2.14 (1.46, 3.12)	0.000	1.89 (1.29, 2.78)	0.001	1.09 (0.47, 2.56)	0.838	1.08 (0.46, 2.54)	0.867
	Northern	0.44 (0.24, 0.81)	0.009	0.57 (0.30, 1.06)	0.076	0.38 (0.08, 1.70)	0.205	0.40 (0.09, 1.81)	0.232
	Upper East	0.45 (0.24, 0.83)	0.010	0.56 (0.30, 1.04)	0.068	0.26 (0.07, 0.91)	0.035	0.27 (0.08, 0.97)	0.045
	Upper West	0.26 (0.14, 0.49)	0.000	0.35 (0.19, 0.68)	0.002	0.09 (0.03, 0.27)	0.000	0.10 (0.03, 0.29)	0.000
	Volta	1.16	0.618	1.39	0.278	0.94	0.931	0.97	0.971
		(0.64, 2.10)		(0.77, 2.53)		(0.21, 4.17)		(0.22, 4.35)	
	Western	0.93 (0.64, 1.35)	0.701	0.88 (0.61, 1.29)	0.519	0.70 (0.28, 1.76)	0.446	0.70 (0.28, 1.76)	0.445
UCV	Before	1 (Ref)		1 (Ref)		1 (Ref)		1 (Ref)	
	After	4.79 (1.53, 15.05)	0.007	2.90 (0.91, 9.23)	0.071	2.53 (0.35, 18.38)	0.358	2.12 (0.29, 15.73)	0.463

A higher proportion of males (6.76%) tested positive for HBV compared with females (3.13%) with the difference being statistically significant. Participants who were 20 years or younger had a lower prevalence (3.16%) whereas those in the age group 31-40 years had the highest prevalence (9.88%). Participants who resided in the Upper West Region had the highest prevalence (19.12%) followed by Northern Region (12.26%) and Upper East (12.04%) compared with those who resided in Greater Accra Region which had the least (2.80%). Participants who were born before the introduction of the UCV against HBV had the highest prevalence (5.24%) compared with those born after the introduction (0.99%).

HCV was more prevalent in males (1.06%) compared with the females (0.78%) although the difference between them was not statistically significant. The differences in the prevalence of HCV in the region of residence was statistically significant with those in the Upper West Region having the highest prevalence of 5.66% and Central Region residents having the least (4.7%). The proportion of participants who were 20 years or younger who tested positive for HCV was 0.8% compared with the highest estimated of 1.23% in the 30-41 year group.

### Predictors of HBV and HCV infections

Crude and adjusted Odds ratios (COR and AOR) were undertaken to determine the predictors of HBV and HCV infection in our study population utilising the multivariable logistics regression modelling. In the multivariable model, males were 3 times more likely to be HBV positive compared to females (AOR=2.77, 95% CI:1.84-4.15, p-value < 0.001). Additionally, Participants who were 21-30 years (AOR = 0.48; 95% CI: 0.37-0.62, p-value < 0.001) and 31-40 years (AOR = 0.45; 95% CI: 0.28-0.73, p-value = 0.001) were 52% and 55% less likely to be HBV-positive compared to those aged and 20 years or below respectively. Compared with participants resident in Central Region, those in Greater Accra Region had increased likelihood of being HBV-positive (AOR = 1.89; 95% CI: 1.29-2.78, p-value = 0.001). On the contrary, those in Upper West Region had lower odds of being HBV-positive (AOR = 0.35; 95% CI: 0.19-0.68, p-value = 0.002).

Regarding the multivariable logistic model with HCV as outcome, residents in Brong Ahafo (AOR = 0.30; 95% CI: 0.12-0.75, p-value = 0.011), Upper East (AOR = 0.27; 95% CI: 0.08-0.97, p=0.045) and Upper West (AOR = 0.10; 95% CI: 0.03-0.29, p-value < 0.001) Regions had lower odds compared with residents in Central Region.

## Discussion

The study was undertaken to estimate the prevalence of HBV and HCV among patients screened at a Ghanaian primary level hospital. This was to enable the team to understand the burden of the infections within the catchment area of the Hospital to operationalise the testing and treatment at the facility. The overall prevalence of HBV was estimated at 5.06%. The prevalence is lower than the national prevalence of 12.3% estimated from a metanalysis of 30 studies conducted nationwide studies from 1995 to 2015 14. The lower prevalence follows previous observation of lowering rates over decades in Ghana^14^. The improving socio-economic status and the introduction of policies targeting infection prevention such as universal childhood vaccination against HBV infection, mandatory antenatal HBV testing and HBV vaccination^7^. A recent nationwide cross-sectional review estimated the prevalence in Ghana at 8.48%^15^. The estimated prevalence from the study is within the uncertainty interval of 4.6-8.5% HBV prevalence rate in Africa region of WHO^16^, but lower than aggregated data from studies in East Africa^17^.

Notably, the prevalence among vaccinated and children born after the introduction of the UCV have been observed to be lower, compared with that in unvaccinated groups in Ghana^18^-^20^. The prevalence of 0.99% in participants born after the introduction of the UCV 2002 was significantly less than those born before its introduction of 5.24%. Additionally, the prevalence in the under 20 age group compared with the over 20 age groups attest to this observation and have also been reported previously in Ghana^18^,^19^ and other countries such as China^21^, United States America^22^, Iran^23^. Irrespective of the positive impact achieved from HBV vaccination policies implemented worldwide, majority of the world's population especially in sub-Saharan Africa remain unvaccinated^24^. Our study revealed that less than a third of the individuals born before the introduction of UCV have had some form of vaccination of which approximately a third of whom did not complete their vaccination schedule. However, due to recall bias, the result might not be conclusive.

The study revealed that males had a prevalence twice more than that of females in the study and were 3 times more likely to test positive for HBsAg. The high prevalence in males was also observed in a study among blood donors in Ghana^25^. A systematic review in East Africa found that males were 8 times more likely to test positive for HBV17. Additionally, studies in Pakistan and Taiwan have observed a higher rate HBV infection among males compared with females^26^,^27^. In Ghana and sub-Saharan Africa where most infections are acquired at younger ages through horizontal transmission dynamics, this result would be expected as culturally, boys rather than girls would regularly be involved in more vigorous outdoor activities and are, therefore, more likely to get infected^28^–^30^.

The stack disparity in health care delivery between the northern regions of Ghana and southern and central regions of Ghana may have inadvertently impacted HBV control in these regions. Interestingly, some studies into viral hepatitis have largely excluded these regions^14^,^25^,^31^,^32^ studies which have been undertaken in the 3 northern regions have reported prevalence greater than 8%. Our study observed that there was high prevalence rate, that is, 12.26%, 12.04% and 19.12% in participants who resided in the Northern, Upper East and Upper West respectively compared with the overall national prevalence of 5.24%. Comparatively, the prevalence for participants residing in Greater Accra, Eastern and Brong Ahafo residents were 2.80%, 3.62% and 3.96% respectively. Previous studies have reported prevalence greater than 8% across the 3 northern regions^33^,^34^.

The availability of direct acting antivirals for treating HCV makes it imperative that screening for

### HCV in the Ghanaian population and offering treatment to the infected has become imperative

The prevalence of HCV infection has been observed to vary widely with two systematic reviews reporting prevalence of 3.0 (range: 0-20.1%) and 3.2% (range: 0.5-8.1%)^9^,^35^. The HCV infection in Ghana has been reported to be highest in high-risk groups such as: men who sleep with men, prostitutes and intravenous drug users^9^,^11^. Thus, it would be expected that the prevalence would be lower in our study compared with those undertaken in high-risk groups. The overall prevalence for HCV infection for the study was observed to be 0.93% which was lower than the prevalence from the 2 systematic reviews but within the ranges for both reviews. The figure is lower compared with seroprevalence of HCV aggregated from metanalyses of some studies previously undertaken in Ghana^9^,^35^. Additionally, the prevalence from this study was lower compared with prevalence reported from studies and systematic reviews in Ethiopia, Cameroun, Togo, Malawi pooled seroprevalence of HCV in Ethiopia, a study among sex workers in Togo and a retrospective descriptive study in Cameroun^36^–^38^, but higher compared with studies undertaken in Malaysia and Iran^31^,^39^. Worryingly, the prevalence among the residents of the 3 northern regions of Ghana HCV was higher compared with southern and central regions. Compared with Central Region, residents of Upper East and Upper West Regions were at statistically significant lesser odds of testing negative to HCV. However, the prevalence observed in our study for the residents of the 3 northern regions was lower than previously reported 6.1% and 12.7% prevalence among blood donors in the Northern Region of Ghana^40^,^41^.

We sought to determine the co-infection rate of HBV-HCV among the participants as these unique population tend to have rapid progression to liver complications^42^. It was observed that the HBV-HCV co-infection seroprevalence rate was less than 1 per 1000 participants in our study. This prevalence is lower, compared with 2.3% rate estimated in a recent study undertaken in the Northern Region of Ghana among blood donors a subgroup who tend to be low risk for HCV infection^40^. The result from our study was comparable to a study in Egypt where the co-infection rate was 7 in 1000 43.

## Conclusion

The burden of HBV and HCV infection remains high, although progress has been made in the reduction of the infection rate, especially for HBV. However, there is high rate of individuals born after the introduction of UCV who are either unvaccinated or have not completed their vaccination schedule. There must be increased advocacy for these individuals to initiate the vaccination process and for those who have not completed the schedule to try and complete it. The burden of HBV and HCV may not be uniform throughout Ghana as the northern sector of the country carries greater burden of disease.

Although the study observed a low prevalence of HBV-HCV coinfection, surveillance for viral hepatitis infection ought to target co-infected individuals and treatment initiated immediately when diagnosed as progression to liver complications is rapid.

## Limitation

Results from this study cannot be generalised as most of the participants were screened in Cape Coast although most of them were not residents of Cape Coast, therefore, the conclusions from this study may not adequately represent the general Ghanaian population. This is especially true pertaining to the reported prevalence rates in the region of residence.

Additionally, the old administrative regions were maintained in this study to make comparisons easier although some of the regions have been redemarcated and renamed.
